# Impact of Cardiovascular Risk Factors on Carotid Intima-Media Thickness and Degree of Severity: A Cross-Sectional Study

**DOI:** 10.1371/journal.pone.0144182

**Published:** 2015-12-04

**Authors:** Lijie Ren, Jingjing Cai, Jie Liang, Weiping Li, Zhonghua Sun

**Affiliations:** 1 Departments of Shenzhen Second People′s Hospital, clinical medicine college of Anhui Medical University, Shenzhen, Guangdong Province, China; 2 Department of neurology, Shenzhen Second People's Hospital, Shenzhen, Guangdong Province, China; 3 Departments of Shenzhen Second People′s Hospital, clinical medicine college of Guangzhou Medical University, Shenzhen, Guangdong Province, China; 4 Department of neurosurgery, Shenzhen Second People's Hospital, Shenzhen, Guangdong Province, China; 5 Department of Medical Radiation Sciences, Curtin University, Perth, Western Australia, Australia; Shenzhen institutes of advanced technology, CHINA

## Abstract

**Objective:**

Age, hypertension, dyslipidemia and diabetes are common cardiovascular risk factors (CVRFs) that contribute to the development of atherosclerosis in cardiovascular system including carotid artery disease. However, the impact of these risk factors on the increased carotid intima-media thickness (cIMT) and degree of carotid severity remains to be further clarified. This study aims to evaluate the relationship between CVRFs and degree of carotid severity and cIMT in high-risk subjects.

**Methods:**

Four thousand and three hundred ninety-four subjects with one or more risk factors were retrospectively reviewed in this study. Patients were divided into different groups based on age, the type and quantity of CVRFs. cIMT and degree of carotid artery stenosis were measured and analyzed based on carotid ultrasound imaging with findings compared to the CVRFs to determine the correlation between these variables.

**Results:**

Aging was significantly associated with degree of severity (P < 0.05) and cIMT was significantly increased with age (P < 0.05). Individual CVRF analysis shows that hypertension was more related to the degree of severity than dyslipidemia and diabetes with corresponding abnormal cIMT rates being 79.39%, 72.98% and 32.37%, respectively. The prevalence of carotid atherosclerosis were 20.06%, 22.88% and 28.63%, respectively corresponding to patients with zero, one and more than one chronic diseases. The percentage of abnormal cIMT in hypertensive patient group with dyslipidemia is significantly higher than the other groups (P< 0.05).

**Conclusions:**

This study shows a direct correlation between the degree of carotid severity and cIMT and cardiovascular risk factors, especially with age and hypertension. Carotid atherosclerosis is closely related to the number of cardiovascular risk factors.

## Introduction

Atherosclerosis constitutes one of the most common causes of stroke, due to embolism from a fissured or ruptured plaque [1]. It is the result of a combination of various physiological processes[2]. Plaque formation is a chronic process which is caused or accelerated by age, hypertension, diabetes and high blood cholesterol [3–6]. Carotid artery intima-media thickness (cIMT) is a measure of subclinical atherosclerosis associated with cardiovascular risk factors (CVRFs) and is predictive of stroke incident [7–11]. Combined cIMT and plaque assessment was considered to be better than either measure alone [12]. Thus, it is clinically important to examine the effects of risk factors on progression of cIMT and atherosclerosis, therefore, achieving the goal of reducing the mortality associated with cerebrovascular events.

Hypertension, diabetes and blood lipid play different role in the formation of plaque and development of cardiovascular disease. Atherosclerosis was caused by a combination of dysfunction of the endothelium of the vessels, oxidative stress and inflammation [2, 13, 14]. A previous study indicated that the cIMT was positively correlated with blood pressure variability in patients with hypertension [15]. A prospective study reported the association of both total serum cholesterol levels and the differences in cholesterol homeostasis with cIMT [16]. In diabetic patients, the latest evidence-based medicine data show a significant increase in IMT [17].

Importantly, the risk of developing atherosclerosis is greatly increased by presence of multiple factors[18]. Epidemiologic studies have stressed the important impact of multiple risk factor profiles on the prediction and prevention of coronary artery disease [19]. Early studies reported the deleterious influence of multiple risk factors on the cIMT in children and young adults [20, 21]. Recently, several studies have confirmed the greater impact of multiple risk factors on cIMT than individual CVRFs on different population groups [22–24], but these studies were limited by a small sample size. Therefore, we conducted this study comprising 4394 subjects with high risk of cardiovascular disease. The aim of this study is to investigate the cIMT in different age groups and explore the relationship between two or more risk factors. It was hypothesized that multiple risk factors have greater impact on cIMT and degree of stenosis, with results providing guidance to clinicians for prevention of carotid atherosclerosis.

## Methods

### Population and Study design

The study was composed of 6 community health centers in Futian District, Shenzhen, China. From November 2012 to March 2013, a total of 14270 residents over 40 years old underwent physical and biochemical examinations as part of their clinical diagnosis of chronic cardiovascular diseases. Exclusion criteria were as follows: (1) subjects with no risk factors; (2) subjects did not agree to have the carotid ultrasonography examination; (3) the examination data was incomplete or missing. Researchers selected the appropriate subjects in the screening process according to inclusion and exclusion criteria. Finally, 4394 subjects met the selection criteria and were included in the analysis. The following details for each patient were collected: age, gender, blood pressure, body mass index (BMI), fasting plasma glucose (FPG), total cholesterol (TC), low density lipoprotein cholesterol (LDL-C), high density lipoprotein cholesterol (HDL-C) and triglyceride (TG). Informed consent forms were obtained from all subjects.

### Definition of risk factors

Definition criterion of risk factors were guided by Adult Treatment Panel III and the World Health Organization [25, 26]. Blood pressure was measured at left arm brachial artery in resting state. Blood pressure was measured by Yuyue mercury sphygmomanometer and NISSEI DS-500 blood pressure monitor. Hypertension was defined as resting systolic blood pressure (SBP) ≥ 140 mmHg, and /or diastolic blood pressure (DBP) ≥ 90 mmHg, or use of antihypertensive drugs. Blood lipid was measured with morning fasting blood, and all the subjects were examined by Cardiochek Portable blood detector. Dyslipidemia was defined as taking antilipemic drugs or having one or more of the following: TC≥5.2 mmol/L, LDL-C≥3.4 mmol/L, HDL-C≤1.0 mmol/L, or TG≥1.70 mmol/L. FPG was detected with morning fasting fingertip blood by Abbott Optium glucose meters. Diabetes mellitus was defined as FPG ≥ 7.0 mmol/L, or use of medication for diabetes. Hypertension, dyslipidemia and diabetes were the major chronic cardiovascular diseases which were associated with increasing the risk of stroke, therefore, comprising the main content of this study.

### Carotid ultrasonography

The carotid ultrasound scan was carried out in the same period of 6 community health centers, with each patient performed by experienced sonographers with more than ten years of experience in ultrasound imaging by using high-resolution ultrasound Doppler system (SSI6000, Kai-Li ultrasound, China). During the data collection, researchers measured the degree of severity and cIMT again based on the ultrasound images. When there was discrepancy in the measurement of degree of severity or cIMT difference was greater than 0.2mm between the two assessors, a third assessor was asked to perform the measurements, and the averaged results of the similar findings were used. All the subjects were measured in a supine position. The scanning was performed on bilateral common carotid arteries, carotid bulb, carotid bifurcation and the origin of the internal carotid artery, with each providing proximal and distal wall data for the right and left carotid arteries. In addition, the scanning was examined at different angles of insonation. IMT was measured from multiple segments of carotid artery with the maximum IMT value used as the final result. When a carotid plaque was present on the artery wall, the maximum value is defined as the maximum height of the plaque. Thus, mean maximum measures represent the plaque burden. The mean maximum values of the common cIMT in the distal wall of the left and right carotid arteries were used as the dependent variables. The presence of plaques and degree of stenosis are characterized according to the Mannheim Consensus 2006 [27]. Carotid artery disease was classified into 4 categories based on the degree of severity (grade 1: IMT <0.9 mm, grade 2: IMT > 0.9 mm, grade 3: presence of carotid plaques with stenosis <50% and grade 4: presence of carotid plaques with stenosis >50%). Carotid IMT <0.9 mm (Grade 1) was regard as normal, while carotid IMT >0.9 mm was regarded as abnormal.

#### Data collection

Each subject has a unique serial number as an identification code. The physical and biochemical examination results and carotid ultrasound measurement data were entered according to the subject’s serial number by a third party (Shenzhen New Element Medical company). To ensure data consistency, two staff members as a group, with one responsible for data entry, another one for the data entry check. All the information was input into Shenzhen Second People′s Hospital stroke screening platform information system.

#### Statistical analysis

Data were analyzed using the software Statistical Package for the Social Sciences (SPSS, version 12.0, USA). To confirm the significance of CVRFs, the models were constructed including the individual risk factors and the possible interactions among these risk factors. To demonstrate the relationship between age and degree of severity, subjects were divided into four groups and the proportion of each grade was calculated. The effect of chronic cardiovascular diseases on atherosclerosis was examined by comparing the grade of severity and mean cIMT values of individuals with 0, 1, 2, and 3 chronic diseases. Risk comparison of continuous variables between groups was performed using x^2^ test. P < 0.05 was considered statistically significant.

## Results

Table 1 shows the patient’s demographic characteristics. There were 2134 males (48.57%) and 2260 females (51.42%) with the mean age 61.21±10.32 years.

**Table 1:** Patient characteristics of study population.

	Category	Subjects (number +percentage)	
**Sex**	Male	2134	48.57%
	Female	2260	51.43%
**Age**	40~50	645	14.68%
	50~60	1373	31.25%
	60~70	1330	30.27%
	>70	1046	23.81%

**Fig 1:**
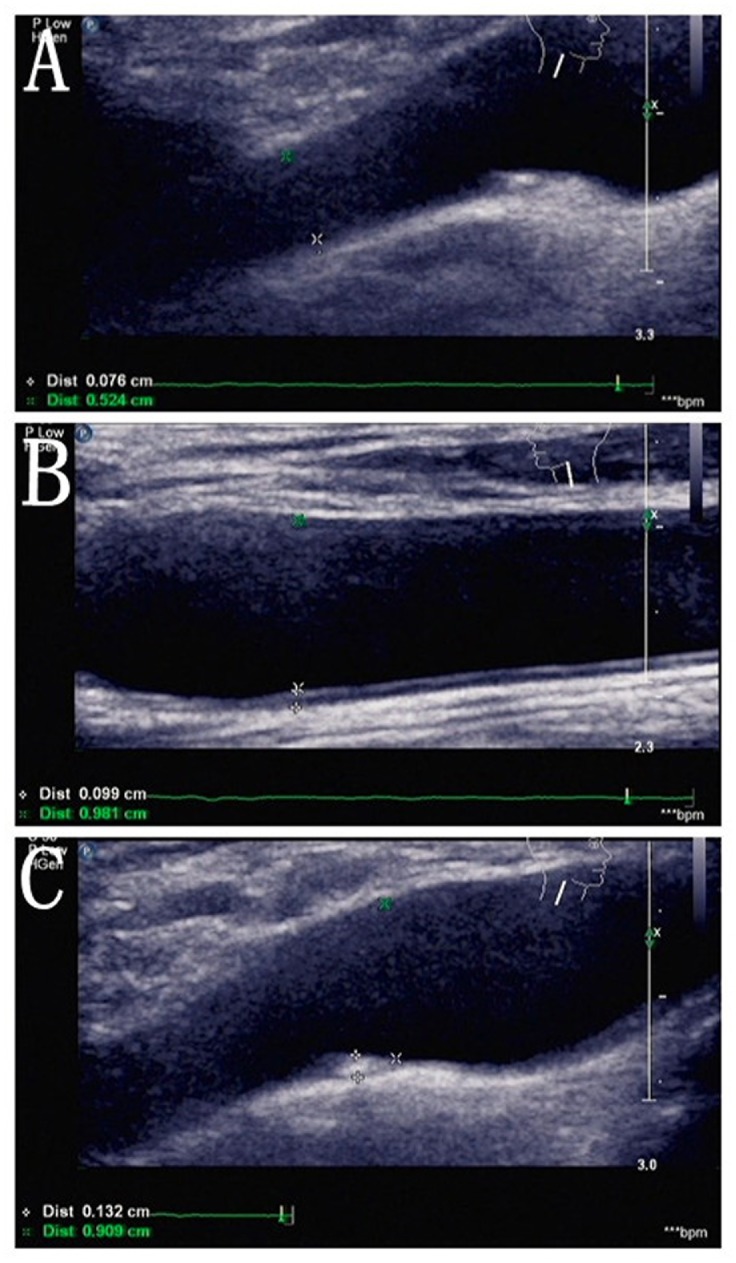
Carotid ultrasound image. *Representing thickening or plaque of carotid artery. (A) Normal carotid intima; (B) Carotid intimal thickening; (C) Carotid artery plaque.

**Fig 2:**
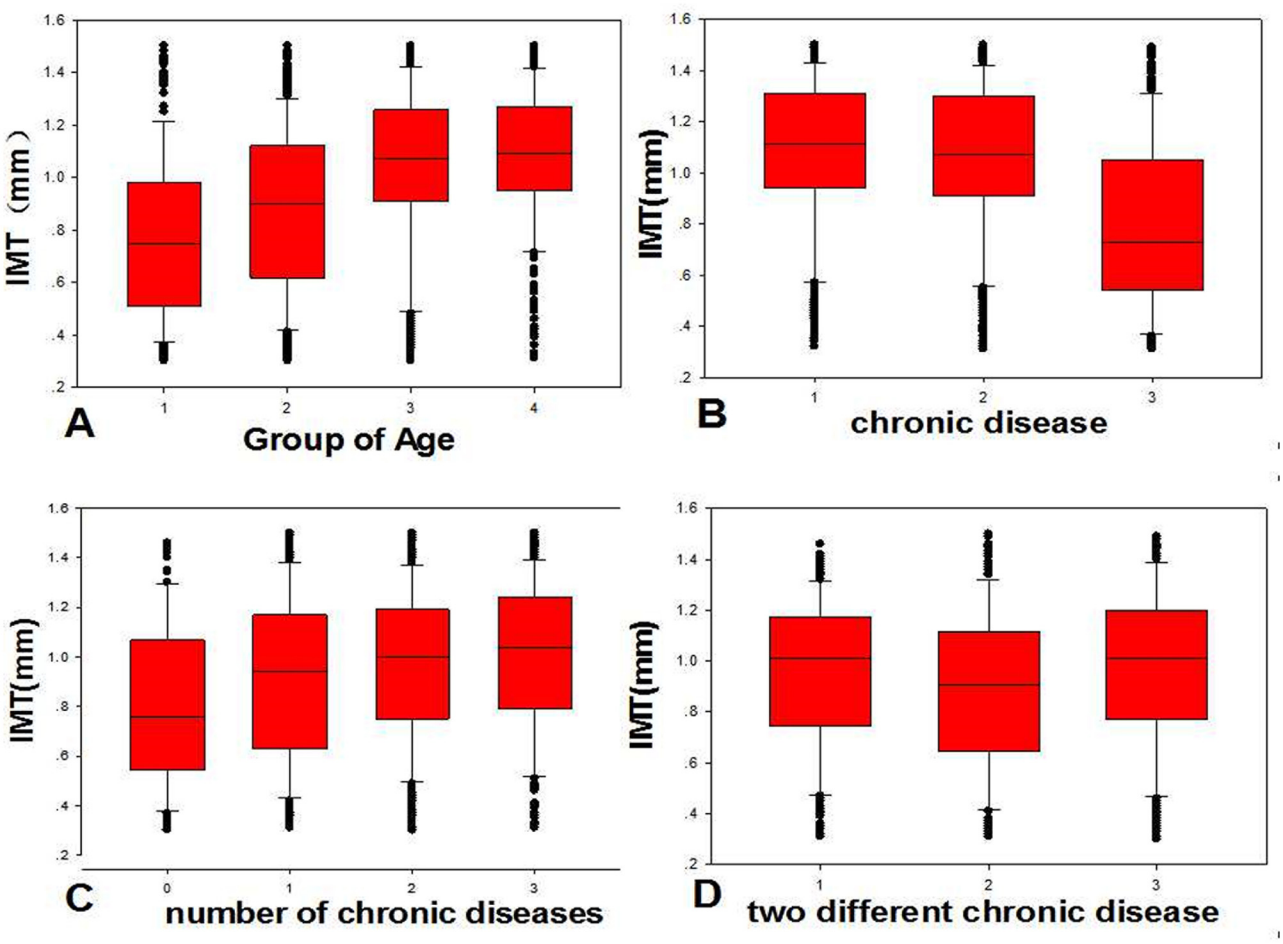
The relation between cIMT and CVRFs. **A** shows the cIMT by the age. Box 1: age 40–50 years; Box 2: age 50–60 years; Box 3: age 60–70 years; Box 4: >70 years. **B** demonstrates the cIMT by the types of chronic disease. Box 1:hypertension; Box 2: dyslipidemia; Box 3: diabetes. **C** shows the cIMT by the number of chronic cardiovascular diseases. Box 0:without chronic cardiovascular disease; Box 1:1 type of chroinc cardiovascular disease; Box 2: 2 types of chronic cardiovascular diseases; Box 3:3 types of chronic cardiovascular diseases. **D** shows the cIMT by two different chronic cardiovascular diseases. Box 1: Combination of hypertension with diabetes; Box 2: combination of dyslipidemia with diabetes; Box 3: combination of hypertension with dyslipidemia.

Table 2 lists the different age groups with carotid ultrasound results. The results showed that cIMT were <0.9 mm in 1711 patients (38.94%), and >0.9 mm in the remaining patients (61.06%). Forty-eight patients were scored with grade 4 consisting of 34 subjects with moderate stenosis, 6 with severe stenosis and 8 with occlusion. In the age group of 40–50 years, abnormal percentage (26.67%) was significantly lower than the other groups (P<0.05). In the age group older than 70 years, 36.04% had arterial plaque formation or stenosis. Fig 1 is an example showing normal carotid endometrium, intimal thickening and carotid plaque. Fig 2A)is the box plot demonstrating the relationship between age and cIMT, with the trend showing that the mean cIMT was increased by age.

**Table 2:** Carotid ultrasound results based on different age groups. Note: * Due to the presence of the desired frequency <5, the use of Fisher's exact test.

Groups	All subjects	Grade 1		Grade 2		Grade 3		Grade 4	
		Subjects	Percent	Subjects	Percent	Subjects	Percent	Subjects	Percent
**40~50**	645	473	73.33%	104	16.12%	66	10.23%	2	0.31%
**50~60**	1373	682	49.67%	468	34.09%	221	16.10%	2	0.15%
**60~70**	1330	406	30.53%	559	42.03%	353	26.54%	12	0.90%
**>70**	1046	150	14.34%	519	49.62%	345	32.98%	32	3.06%
**Total**	4394	1711	38.94%	1650	37.55%	985	22.42%	48	1.09%
**X2**	~			502.649		461.132		176.137*	
**P**	~			0		0		0.000*	

Table 3 shows the relationship between different chronic cardiovascular diseases and carotid ultrasound analysis. The number of subjects with hypertension accounted for 60.28% of the total population. In the hypertension group, the number of abnormal cIMT patients (79.39%) was higher than the other groups (72.98% and32.37%). In the dyslipidemia group, the subjects of grade 2 and grade 3 were greater than the diabetic group. Fig 2B shows the relationship between different chronic cardiovascular diseases and cIMT, with the mean cIMT measurement highest in the hypertension group.

**Table 3:** The relationship between different chronic cardiovascular diseases and degrees of severity. Note: * Due to the presence of the desired frequency <5, the use of Fisher's exact test.Table 4 shows the relationship between number of chronic cardiovascular diseases and degree of severity. In the group with no chronic disease, cIMT was normal in more than half of the subjects. With the increasing number of chronic cardiovascular disease, the exposure rate of subject’s intimal thickening, plaque formation and moderate carotid stenosis increased. Fig 2C shows the cIMT in subjects with different numbers of chronic cardiovascular diseases with significant difference noticed between groups (p<0.05).

Groups	All Subjects	Grade 1		Grade 2		Grade 3		Grade 4	
		Subjects	Percent	Subjects	Percent	Subjects	Percent	Subjects	Percent
**Hypertension**	2649	546	20.61%	1059	39.96%	1003	37.87%	41	1.55%
**Dyslipidemia**	2624	709	27.02%	961	36.64%	918	35.00%	36	1.37%
**Diabetes**	1146	775	67.63%	182	15.89%	170	14.86%	19	1.66%

**Table 4:** Relationship between the number of chronic disease and degrees of severity. Note: * Due to the presence of the desired frequency <5, the use of Fisher's exact test.

Number of chronic disease	All subjects	Grade1		Grade2		Grade 3		Grade 4	
		Subjects	Percent	Subjects	Percent	Subjects	Percent	Subjects	Percent
**0**	223	131	58.74%	56	25.11%	27	12.11%	1	0.45%
**1**	1316	573	43.54%	434	32.98%	264	20.06%	7	0.53%
**2**	2146	806	37.56%	839	39.10%	491	22.88%	31	1.44%
**3**	709	201	28.35%	321	45.28%	203	28.63%	9	1.27%
**Total**	4394	1711	38.94%	1650	37.55%	985	22.42%	48	1.09%
**X** ^**2**^	~	~		76.433		68.509		11.908*	
**P**	~	~		0		0		0.008*	

Table 5 lists the relationship between two different chronic cardiovascular disease and degree of severity. A total of 2146 subjects suffered from two types of chronic cardiovascular diseases at the same time. Subjects with hypertension and dyslipidemia accounted for 79.40% of the total abnormal cases. Fig 2D shows the box plot of two different chronic cardiovascular diseases and cIMT. Findings suggested that the collective effect of hypertension and dyslipidemia should be underscored. Two different chronic cardiovascular diseases and ultrasound results were statistically significant different between the combined groups consisting of different chronic cardiovascular diseases (p <0.05).

**Table 5:** Relationship between two different chronic cardiovascular diseases and degrees of severity. Note: * Due to the presence of the desired frequency <5, the use of Fisher's exact test.

Groups	All subjects	Grade 1		Grade 2		Grade 3		Grade 4	
		Subjects	TotalPercent	Subjects	Abnormal Percent	Subjects	Abnormal Percent	Subjects	Abnormal Percent
**Hypertension+diabetes**	231	65	28.14%	112	8.36%	62	4.63%	7	0.52%
**Dyslipidemia+diabetes**	206	96	46.60%	65	4.85%	34	2.54%	1	0.07%
**Hypertension+dyslipidemia**	1709	645	37.74%	662	49.40%	395	29.48%	23	1.72%
**Total**	2146	806	37.56%	839	62.61%	491	36.64%	31	2.31%
**X** ^**2**^	~	~		20.176		14.062		7.533*	
**P**	~	~		0		0.001		0.017*	

## Discussion

The present study shows that middle-aged and older adults with CVRFs display increased cIMT and higher grade of severity than the younger age groups. In addition, this study shows age, blood pressure, blood lipid and fasting plasma glucose are independent predictors of carotid atherosclerosis. Carotid IMT measurements have been applied in the study of cardiovascular disease for more than 2 decades [28]. Ultrasound measurements of cIMT and carotid plaque have been widely accepted as a noninvasive method to assess the extent of cerebrovascular disease [8, 9, 29]. Assessment of carotid artery vessels is increasingly used as the focus of carotid artery ultrasound imaging for cardiovascular risk prediction [12]. In this study, the ultrasound results of the carotid arteries were evaluated and correlated with the CVRFs for atherosclerosis. Carotid IMT was tested and degrees of severity were divided into four grades to enable us perform a comprehensive analysis of the cardiovascular risk factors in relation to the carotid dimensional changes. Results of this study further clarified the relationship between conventional risk factors (age, hypertension, dyslipidemia and diabetes) and cIMT in a large number of middle-age and older Chinese populations.

Total cholesterol, smoking and systolic blood pressure has been proved to be strong long-term predictors of total plaque area [30]. Individual components of CVRFs play a different role in the formation of plaque. Koskinen et al. evaluated the 6-year progression of cIMT in 1809 subjects aged 27–35 years, and the study found that conventional risk factors predicted cIMT progression in young adults [31]. This finding indicates that atherosclerotic change to the carotid artery already exists in an early stage, which is a preclinical phase of the disease. The proportion of older residents is accompanied by a significant increase in chronic cardiovascular disease, and this may lead to increased cIMT. However, Lee et al. considered vascular aging was an independent risk factor for cardiovascular disease and aging was a major determinant of arterial stiffness and IMT [32]. Sun et al. suggested that vascular aging itself is an independent risk factor for atherosclerosis [33]. Results of this study are consistent with these findings, further highlight that aging is an independent risk factor of atherosclerosis.

Hypertension was found to be associated with an increased risk of neurological events, according to an early report [34]. Waldstein et al. found in combination with other risk factors, hypertension results in higher risk of stroke [35]. Rundek et al. demonstrated a larger cIMT in adolescents suffering from hypertension than healthy controls [36]. The mechanisms of association between hypertension and atherosclerosis may be linked with angiotensin II, which was a pro-inflammatory and pro-oxidant stimulus[37]. The results are consistent with the INTERHEART study [38], which reported that the population attributable risks (PAR) of hypertension is 17.9% higher than diabetes (PAR 9.9%). Atherosclerosis has been shown to be associated with high cholesterol level [39]. The mean serum cholesterol among Chinese population is lower than that of Western standards [40], however, serum cholesterol was directly related to coronary heart disease mortality even at relatively low levels [41]. Our research and previous studies both indicate dyslipidemia is still a risk factor of arteriosclerosis among the Chinese people.

Diabetes is one of the major risk factors associated with carotid atherosclerosis [42]. It has been reported that patients with obesity or diabetes tend to have thicker and stiffer carotid arteries and are more likely to suffer from cerebrovascular events [43]. Although our study shows the influence of diabetes is lower than hypertension and dyslipidemia, a cross-sectional population study proved glycemic status was associated with all grades of carotid atherosclerosis [44]. Thus, patients with diabetes should receive preventive interventions to reduce the stroke risk.

Although the relationship between individual risk factors and cIMT has been proved, the interaction of two or more risk factors cannot be ignored. In this study, we found that two or more different risk factors are independent of individual factors that have an impact on cIMT (Tables 4 and 5). This reminds us of underscoring the interactions between multiple CVRFs. Chuang et al. concluded that hypertension and dyslipidemia with type 2 diabetes have cumulative effects on the burden of carotid plaque[45]. In a study of 518 subjects of the same age-race-gender distribution, Urbina et al. examined the healthy young adults, and their results showed the trend of increasing cIMT at different carotid segments with increasing number of risk factors [20]. Paul et al. found the odds ratio for patients with >3 risk factors versus no risk factors having cIMT in the top fifth percentile was 4.7 (p = 0.01) [46]. Lili et al. in their comparative study concluded that three or more CVRFs have a greater impact on cIMT and elasticity than two or less CVRFs [23], but the cIMT data were taken from only left common carotid artery, thus their results may underestimate the relationships between multiple CVRFs. The mechanism of combined effect of two risk factors and interaction of multiple risk factors needs to be confirmed in future studies.

In addition to the influence of conventional risk factors on carotid atherosclerosis, the impact of other risk factors is attracting attention in the literature. Accumulating evidence indicates that psychological factors contribute to morbidity and mortality in cardiovascular disease. The INTERHEART study shows PAR of psychosocial factor is 32.5% even higher than hypertension [38]. Previous studies have shown depression or anxiety was associated with an increased cIMT and subclinical arteriosclerosis [47–49]. A meta-analysis has provided strong evidence that depression is a significant risk factor for stroke[50]. Our study did not include the psychological factors, and a further study will explore the relationship between cIMT and psychological factors. Tatjana et al. found vascular risk factors explain only 11% of variance in cIMT [51], this study emphasizes the importance of novel genetic and environmental factors in underlying unexplained subclinical atherosclerosis. There are still some controversies in the risk factors of increased cIMT, thus, further research requires multiple corners, population-based and large number subjects to verify these early results.

This study verifies several findings. First, the study indicates that high-risk middle-aged and older population has a high proportion of carotid atherosclerosis. Second, aging was significantly associated with increased cIMT. Third, hypertension, dyslipidemia and diabetes plays an important role in the process of developing atherosclerosis and the impact of hypertension on cIMT is higher than dyslipidemia and diabetes. Fourth, the more risk factors and more chronic cardiovascular diseases that patients have, the higher risk of developing atherosclerosis.

Some limitations exist in this study that should be addressed. The study design involved four traditional CVRFs: age, hypertension, diabetes and dyslipidemia. It did not include smoking, homocysteine, psychological factors and other CVRFs. Therefore, further research is needed to allow robust conclusion to be drawn. In addition, our study divided the groups according to age, the type of CVRFs and the quantity of CVRFs. We did not stratify risk factors based on biochemical outcomes. This might underestimate the impact of individual risk factors.

## Conclusions

In conclusion, this study shows that age, hypertension, dyslipidemia and diabetes plays a different role in the formation of plaque and the combination effect is increased with the burden of atherosclerosis. Among these factors, hypertension shows the highest impact on cIMT compared to dyslipidemia and diabetes. The degree of carotid severity and cIMT is increased with the number of chronic cardiovascular disease. This finding indicates the need for prevention and control of atherosclerosis with a focus on hypertension and multiple CVRFs.
